# Rotational head acceleration and traumatic brain injury in combat sports: a systematic review

**DOI:** 10.1093/bmb/ldac002

**Published:** 2022-01-28

**Authors:** Kabir Singh Lota, Nikos Malliaropoulos, Wiesław Blach, Takeshi Kamitani, Akira Ikumi, Vasileios Korakakis, Nicola Maffulli

**Affiliations:** Barts and The London School of Medicine and Dentistry, London, E1 2AD, UK; Centre for Sports and Exercise Medicine, Queen Mary University of London, E1 4DG, UK; Centre for Sports and Exercise Medicine, Queen Mary University of London, E1 4DG, UK; Sports and Exercise Medicine Clinic, Asklipiou 17, 54639 Thessaloniki, Greece; Rheumatology Department, Sports Clinic, Barts Health NHS Trust, London, E1 4DG, UK; Department of Physical Education and Sport, University School of Physical Education, Wrocław 51-612, Poland; School of Sport and Health Science, Tokai Gakuen University, 21-233 Nishinohora, Ukigai, Miyoshi, Aichi, 470-0207, Japan; Department of Orthopedic Surgery and Sports Medicine, Tsukuba University Hospital Mito Clinical Education and Training Center, 3-2-7 Miyamachi, Mito, Ibaraki 310-0015, Japan; Aspetar Orthopaedic and Sports Medicine Hospital, 29222, Doha, Qatar; Centre for Sports and Exercise Medicine, Queen Mary University of London, E1 4DG, UK; Department of Medicine, Surgery and Dentistry, University of Salerno, Via S. Allende, Baronissi, Salerno 84081, Italy; School of Pharmacy and Bioengineering, Faculty of Medicine, Keele University, Stoke-on-Trent, ST4 7QB, UK

**Keywords:** acceleration, brain injuries, traumatic, head, humans, sports

## Abstract

**Background:**

Traumatic brain injury (TBI) in combat sports is relatively common, and rotational acceleration (RA) is a strong biomechanical predictor of TBI. This review summarizes RA values generated from head impacts in combat sport and puts them in the context of present evidence regarding TBI thresholds.

**Sources of data:**

PubMed, EMBASE, Web of Science, Cochrane Library and Scopus were searched from inception to 31^st^ December 2021. Twenty-two studies presenting RA data from head impacts across boxing, taekwondo, judo, wrestling and MMA were included. The AXIS tool was used to assess the quality of studies.

**Areas of agreement:**

RA was greater following direct head strikes compared to being thrown or taken down. RA from throws and takedowns was mostly below reported injury thresholds. Injury thresholds must not be used in the absence of clinical assessment when TBI is suspected. Athletes displaying signs or symptoms of TBI must be removed from play and medically evaluated immediately.

**Areas of controversy:**

Methodological heterogeneity made it difficult to develop sport-specific conclusions. The role of headgear in certain striking sports remains contentious.

**Growing points:**

RA can be used to suggest and assess the effect of safety changes in combat sports. Gradual loading of training activities based on RA may be considered when planning sessions. Governing bodies must continue to work to minimize RA generated from head impacts.

**Areas timely for developing research:**

Prospective research collecting real-time RA data is required to further understanding of TBI in combat sports.

## Introduction

Traumatic brain injury (TBI) occurs when external forces transmitted to the head cause neuropathologic damage and/or dysfunction.^1^ Examples of TBI include concussion, diffuse axonal injury (DAI) and acute subdural haematoma (ASDH). TBI is classified as mild, moderate or severe. Clinical signs vary and include new-onset loss of consciousness (LOC), post-traumatic amnesia (PTA), alterations in mental state (e.g. confusion) and focal neurological deficit.^2^ Most athletes recover fully within one week of TBI, yet there is growing evidence of long-term neurological sequelae including recurrent headache, cognitive impairment and death.^1^

TBI is common in combat sports, with approximately 300 000 cases diagnosed every year.^3^ Combat sports are generally of a striking (boxing, taekwondo) or grappling (judo, wrestling) nature, with mixed martial arts (MMA) combining multiple disciplines into one sport that includes both striking and grappling. TBI can therefore occur following direct head strikes or after being thrown or taken down. The rapid acceleration–deceleration forces generated upon impact cause vigorous movements of the brain within the skull, resulting in widespread neuronal and vascular damage in proportion to the degree of acceleration experienced.^4^ Acceleration can be linear or rotational, depending on head movement relative to the body. Although both types can be present during an impact, rotational acceleration (RA) is more strongly implicated in TBI.^5^ Brain tissue tends to deform following the application of shearing forces, and approximately 90% of the total shearing stresses produced can be attributed to RA.^6^

TBI is likely underdiagnosed for several reasons, including the absence of a mandatory injury-reporting system and the lack of more typical signs at presentation.^7^ The confusion surrounding its diagnosis has led to inconsistent management, with athletes returning inappropriately early to sport, thus increasing the risk of further TBI.^7^^,^^8^ Various RA thresholds have been suggested for injuries such as concussion (4500 rad/s^2^), DAI and ASDH (10 000 rad/s^2^).^4^^,^^9^^,^^10^ We systematically reviewed the available data on RA generated from combat sport impacts to the head and put these in the context of proposed TBI thresholds.

## Methods

The search strategy and reporting of this review was conducted in accordance with the Preferred Reporting Items for Systematic Reviews and Meta-Analyses (PRISMA) guidelines. The review protocol was registered with the International Prospective Register of Systematic Reviews (PROSPERO 2020 CRD42020216470).

### Eligibility criteria

Studies were considered eligible if they were published in English and measured RA (rad/s^2^) produced in head impacts during combat sport. Examples of combat sports include boxing, kickboxing, karate, taekwondo, judo, wrestling and MMA. No restrictions on study type or methodology were set; however, animal studies and studies involving participants younger than 16 years of age were excluded, as injury thresholds had been developed only for adults. Studies that exclusively measured linear acceleration, or any other kinematic variable that was not RA, were also excluded. Other systematic reviews were not eligible, although the reference lists of reviews identified through the literature search were screened for potentially relevant studies. Additional details (i.e. use of headgear, associated injury/LOC, competition/sparring) were recorded if present. The inclusion and exclusion criteria are shown in Table 1.

**Table 1:** Inclusion and exclusion criteria

Inclusion	Exclusion
• Measured RA (rad/s^2^) generated from combat sport impacts to the head • Adult participants (≥16 years old) • Any means of data collection • Written in English and published in peer-reviewed journals	• Measured RA of other parts of the body • Non-combat sports • Measured kinematic variables other than RA • Other systematic reviews • Not available in English • Animal studies

### Search strategy and data sources

A comprehensive electronic literature search was performed independently by two researchers across PubMed, EMBASE, Web of Science, Cochrane Library and Scopus on December 31, 2021, with all results shown from inception. Boolean operators were also used to broaden the search. A full breakdown of the search strategy is shown in Table 2.

**Table 2:** Full breakdown of terms used in literature search

(boxing OR boxers OR kickbox^*^ OR ‘muay thai’ OR karate OR taekwondo OR judo OR wrestl^*^ OR ‘martial art^*^’ OR ‘combat sport^*^’ OR ‘mixed martial arts’ OR MMA) AND	(rotation^*^ OR angular OR accel^*^ OR biomechanic^*^ OR kinematic^*^ OR movement^*^ OR impact^*^ OR device^*^ OR punch^*^ OR kick^*^ OR strike^*^ OR throw^*^ OR takedown^*^ OR technique^*^) AND	(head OR ‘head impact^*^’ OR brain OR injur^*^ OR traum^*^ OR ‘traumatic brain injur^*^’ OR TBI OR concussion)

The search results were imported into Mendeley reference management software V1.19.4 (Elsevier, New York, New York, USA) with duplicate records identified and removed. The remaining studies were imported into Rayyan (QCRI, Doha, Qatar) for subsequent title and abstract screening. Screening was performed independently by the same two reviewers. The full text of the relevant articles was retrieved for further assessment, and studies that met the eligibility criteria were included. Any disagreements regarding the eligibility of a study were resolved by consensus and involved the opinion of a third researcher.

### Data extraction and analysis

Data extracted from eligible studies included author, year, country, study design, sport investigated, data collection method, technique/location of impact and peak/average peak (±SD) RA. Additional details or comparators were recorded if present. Head impact locations were grouped into ‘front’ (and forehead), ‘side’, ‘top’, ‘back’ and ‘jaw’ in line with research in this field.^11^ The higher value of studies reporting multiple impacts to the same location (i.e. left and right ‘side’) was always taken. One reviewer (KSL) extracted the data and a second reviewer (NM) independently verified the data. Meta-analysis was not possible given the heterogeneity between studies. A descriptive analysis of the data was performed with reference to the proposed TBI thresholds.

### Quality assessment

The same two reviewers independently assessed the methodological quality of each investigation using the Appraisal tool for Cross-Sectional Studies (AXIS).^12^ The AXIS tool consists of 20 items and serves as a means of critical appraisal by addressing study designs, reporting quality and risk of bias.^12^ Four items (3, 7, 13, 14) were removed from scoring across all studies as it was not appropriate to assess non-response.^12^ Further three items (5, 6, 20) were removed from studies involving non-human participants.^12^^,^^13^ Scores were reported as percentages to account for the changes made to the scoring system. Studies were given one point for items met and none for items not met (or where this was unclear). Interpretation of overall study quality was left at the discretion of the research team. Disagreements between authors were resolved by consensus and no study was excluded based on quality assessment.

## Results

### Study selection

**Fig. 1:**
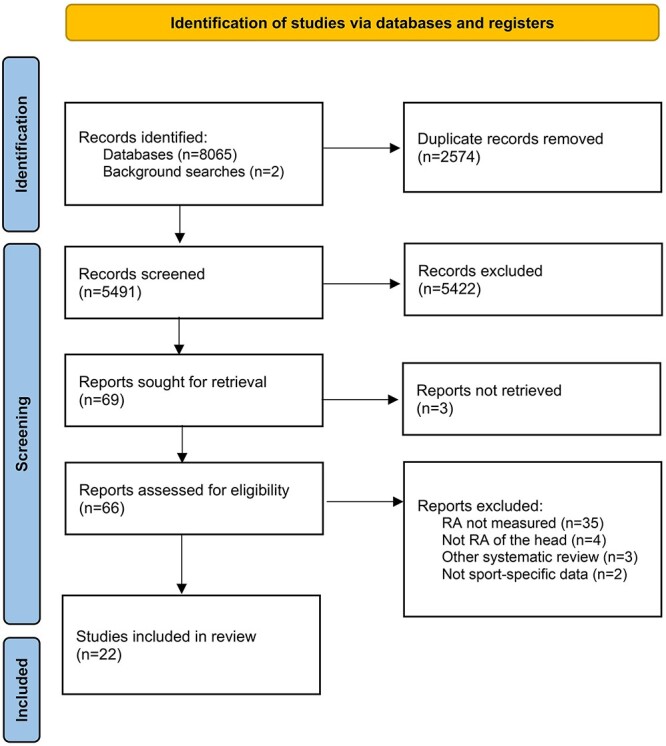
PRISMA flow chart detailing study selection process.

The initial literature search identified 8065 studies, and 5491 remained following duplicate removal. Additional two studies were identified during background searches. Title and abstract screening excluded 5422 studies and left 69 for full-text screening. Of these, 22 studies met the inclusion criteria. The most common reason for exclusion was RA not being measured (*n* = 35). No further studies were identified from screening the reference lists of relevant reviews identified during the literature search. A full breakdown of the selection process can be seen in Figure 1.

### Study characteristics

Studies were published over a 17-year period between 2005 and 2021. Nine studies were from USA^14–22^, five from Japan^23–27^, two each from Korea^28^^,^^29^, Iran^30^^,^^31^ and Ireland^32^^,^^33^ and one each from Canada^34^ and Australia^35^. The combat sports investigated included boxing^14^^,^^15^^,^^18–22^^,^^28^^,^^31^^,^^34^^,^^35^, judo^23–27^, taekwondo^16^^,^^28–30^, MMA^14^^,^^22^^,^^32^^,^^33^ and wrestling^17^. All studies were cross-sectional except for six, which were prospective cohort studies^17^^,^^18^^,^^22^^,^^29^^,^^32^^,^^33^. The studies included data collected on athletes or dummies and involved RA imparted by athletes or mechanical impactors. Two studies^30^^,^^31^ collected data using computer simulation. The remainder were given a code based on their methodology: ‘human-human’ (HH), ‘human-dummy’ (HD) or ‘mechanical-dummy’ (MD). HH studies^17^^,^^18^^,^^21^^,^^22^^,^^24^^,^^25^^,^^29^^,^^32^^,^^33^ involved athletes impacting other athletes who were wearing an accelerometer, fitted as part of skin-patches^17^^,^^25^^,^^29^, headgear^18^^,^^21^^,^^24^ or mouthguards^22^^,^^32^^,^^33^. HD studies^16^^,^^19^^,^^20^^,^^23^^,^^26^^,^^27^ involved athletes impacting dummies fitted with accelerometers. The Hybrid III 50th percentile male anthropometric device (ATD) (175 cm, 77.7 kg) was used in four of these studies^16^^,^^19^^,^^20^^,^^23^ and the POLAR ATD (175 cm, 75 kg) in the remaining two. MD studies^14^^,^^15^^,^^28^^,^^34^^,^^35^ involved mechanical devices (e.g. pendulum) impacting dummies at a given velocity. The Hybrid III 50th percentile male ATD was used in all MD studies. Study characteristics and outcomes are presented in Table 3.

**Table 3:** Study characteristics and outcomes

Author, year	Country	Sport	Method	QA (%)	Headgear (Y/N)	Technique/impact location	RA (rad/s^2^)
Bartsch et al, 2012^14^	USA	Boxing and MMA	MD	100	Y (in some)	Hook punch	Boxing: 1740 (HG), 5240 (no HG) MMA: 5550 (no HG)
Beckwith et al, 2007^15^	USA	Boxing	MD	92	Y	1) Front 2) Side	1) 1530 2) 1753
Boroushak et al, 2018^30^	Iran	Taekwondo	Computer simulation	77	Y	Roundhouse kick	5908 (peak), 2539 (average)
Boroushak et al, 2021^31^	Iran	Boxing	Computer simulation	77	N	Straight punch	4036 (peak), 1140 (average)
Cournoyer et al, 2019^34^	Canada	Boxing	MD	100	N	Hook punch, jab punch	LOC: 11 279.5 ± 4743.1 Non-LOC: 6145.5 ± 4523.5
Doan et al, 2021^21^	USA	Boxing	HH	94	Y	1) Front 2) Side 3) Top 4) Back	1) 1647.38 ± 892.16 2) 1772.52 ± 1031.28 3) 1457.02 ± 544.97 4) 1933.10 ± 1329.29
Fife et al, 2018^16^	USA	Taekwondo	HD	100	Y	1) Hook kick 2) Turning kick 3) Hook punch 4) Jab punch 5) Straight punch	1) 9756 ± 3842 (8483–11 028) 2) 10 927 ± 8017 (8271–13 582) 3) 8712 ± 3685 (7491–9932) 4) 8077 ± 1760 (7493–8660) 5) 9556 ± 2378 (8768–10 343)
Hecimovich et al, 2016^17^	USA	Wrestling	HH	100	Y	1) Front 2) Side 3) Top 4) Back	1) 1698.8 (1027.2–2329.8) 2) 1721.6 (1166.2–2774.0) 3) 2072.8 (1882.1–3373.6) 4) 2434.4 (1630.8–3536.2)
Hitosugi et al, 2014^23^	Japan	Judo	HD	88	N	1) Osoto-gari 2) Ouchi-gari	1) 3315 ± 168 2) 1328 ± 201
Ishikawa et al, 2018^24^	Japan	Judo	HH	94	Y	1) Osoto-gari 2) Ouchi-gari 3) Tai-otoshi 4) Seoi-nage	1) 693.2 2) 401.6 3) 368.3 4) 276.2
Jansen et al, 2021^22^	USA	Boxing and MMA	HH	94	Y (boxing, MMA sparring)	All head impacts	Boxing: Competition: 1642 (1338–2298) (M) Sparring: 1534 (1221–2078) (M), 2019 (1457–2479) (F) MMA^*^: Competition: 3773 (3103–4658) (M) Sparring: 1766 (1359–2373) (M), 1796 (1323–2822) (F)
McIntosh et al, 2015^35^	Australia	Boxing	MD	92	Y (in some)	1) Front 2) Side 3) Jaw	1) 4335 ± 189 (HG), 8365 ± 1900 (no HG) 2) 4323 ± 244 (HG), 7411 ± 812 (no HG) 3) 7173 ± 506 (HG), 8605 ± 113 (no HG)
Murayama et al, 2020^25^	Japan	Judo	HH	100	N	Osoto-gari	679.4 ± 173.6
Murayama et al, 2020^26^	Japan	Judo	HD	100	N	Seoi-nage	1890.1 ± 1151.9 (641.3–3195.4)
Murayama et al, 2014^27^	Japan	Judo	HD	100	N	1) Osoto-gari 2) Ouchi-gari	1) 4572.6 ± 357.4 (UM), 5081.3 ± 691.8 (no UM) 2) 2176.0 ± 826.6 (UM), 1960.0 ± 280.1 (no UM)
O’Sullivan et al, 2016^28^	Korea	Boxing and taekwondo	MD	85	Y	1) Front 2) Side	Boxing: 1) 14065 ± 8502 2) 4427 ± 590 Taekwondo: 1) 20519 ± 5037 2) 8703 ± 3287
O’Sullivan et al, 2017^29^	Korea	Taekwondo	HH	94	Y	All head impacts	22 561 (peak), 4455 ± 3516 (average)
Stojsih et al, 2010^18^	USA	Boxing	HH	94	Y	1) Front 2) Side 3) Top 4) Back	1) 2307 ± 1587 2) 2898 ± 1874 3) 2251 ± 1410 4) 3004 ± 1982
Tiernan et al, 2020^32^	Ireland	MMA	HH	88	N	All head impacts	Concussion: 7560.8 ± 3437 No injury (competition): 7069.5 ± 1277 No injury (sparring): 5055.7 ± 1374
Tiernan et al, 2020^33^	Ireland	MMA	HH	88	N	All head impacts	Concussion: 7561 ± 1825 No injury: 5169 ± 3843
Viano et al, 2005^19^	USA	Boxing	HD	88	N	1) Hook punch 2) Uppercut punch 3) Front 4) Jaw	1) 9306 ± 4485 2) 3181 ± 1343 3) 5452 ± 2107 4) 6896 ± 2848
Walilko et al, 2005^20^	USA	Boxing	HD	94	Y	Front	6344 ± 1789

HH = human–human, HD = human–dummy, MD = mechanical–dummy, HG = headgear, UM = under mat, LOC = loss of consciousness, M = male, F = female.

All RA values were peak/average peak (±SD) measurements, unless otherwise stated.

^*^MMA gloves were used in competition, and boxing gloves were used in sparring.

#### Boxing

Eleven studies^14^^,^^15^^,^^18–22^^,^^28^^,^^31^^,^^34^^,^^35^ measured RA from head impacts in boxing. Five studies were MD^14^^,^^15^^,^^28^^,^^34^^,^^35^, three were HH^18^^,^^21^^,^^22^, two were HD^19^^,^^20^ and one was a computer simulation^31^. Headgear was used in all but three studies^19^^,^^31^^,^^34^. Four studies^14^^,^^19^^,^^31^^,^^34^ assessed RA according to the striking technique used. These included the hook^14^^,^^19^ (1740–9306 rad/s^2^), jab^34^, straight^31^ (4036 rad/s^2^) and uppercut^19^ (3181 rad/s^2^) punches. The RA of hooks and jabs resulting in LOC (11279.5 rad/s^2^) was noticeably higher than those that did not (6145.5 rad/s^2^).^34^ Seven studies^15^^,^^18–21^^,^^28^^,^^35^ assessed RA by location of impact. The highest RA recorded was in front impacts^15^^,^^18–21^^,^^28^^,^^35^ (1530–14 065 rad/s^2^), followed by jaw^19^^,^^35^ (6896–8605 rad/s^2^), side^15^^,^^18^^,^^21^^,^^35^ (1753–4427 rad/s^2^), back^18^^,^^21^ (1933.10–3004 rad/s^2^) and top^18^^,^^21^ (1457.02–2251 rad/s^2^) impacts. RA was similar in competition (1642 rad/s^2^) and sparring (1534 rad/s^2^) when compared.^22^

#### Judo

Five studies^23–27^ measured RA from head impacts in judo. Three studies were HD^23^^,^^26^^,^^27^ and two were HH^24^^,^^25^. Headgear was used in only one study^24^. Four throws were assessed: osoto-gari^23–25^^,^^27^ (679.4–5081.3 rad/s^2^), ouchi-gari^23^^,^^24^^,^^27^ (401.6–2176.0 rad/s^2^), tai-otoshi^24^ (368.3 rad/s^2^) and seoi-nage^24^^,^^26^ (276.2–1890 rad/s^2^). The use of an additional under-mat lowered RA from 5081.3 to 4572.6 rad/s^2^ in osoto-gari.^27^ However, in ouchi-gari, RA increased from 1960.0 to 2176.0 rad/s^2^ when an under-mat was used.^27^

#### Taekwondo

Four studies^16^^,^^28–30^ measured RA from head impacts in taekwondo. Methodologies varied between HH^29^, HD^16^, MD^28^ and computer simulation^30^. Headgear was used in all studies. Two studies^16^^,^^30^ assessed RA by the striking technique used. These included three kicks (roundhouse, hook, turning) and three punches (hook, jab, straight), of which the turning kick^16^ (10 927 rad/s^2^) and straight punch^16^ (9556 rad/s^2^) generated the greatest RA. Two studies^28^^,^^29^ assessed RA by location of impact. RA from side impacts (8703 rad/s^2^) was greater than that from front impacts (4427 rad/s^2^).^28^ For all head impacts, a peak RA of 22 561 rad/s^2^ was recorded.^29^

#### M‌MA

Four studies^14^^,^^22^^,^^32^^,^^33^ measured RA from head impacts in MMA. Three studies were HH^22^^,^^32^^,^^33^ and the other was MD^14^. Headgear was only used during sparring in one study.^22^ The hook punch was the only technique assessed and generated an acceleration of 5550 rad/s^2^ when the impacting device was fitted within an MMA glove.^14^ RA from head impacts diagnosed with concussion (7560.8–7561 rad/s^2^) was higher than from those that were not (5055.7–7069.5 rad/s^2^), whether in competition or during sparring.^32^^,^^33^ RA in competition (3773 rad/s^2^) was greater than in sparring (1766 rad/s^2^), although headgear was not used in competition.^22^

#### Wrestling

One HH study^17^ measured RA from head impacts in wrestling. Headgear was used, and four impact locations (front, side, top, back) were assessed. No specific techniques were investigated. RA was greatest following impact to the back of the head (2434.4 rad/s^2^), followed by the top (2072.8 rad/s^2^), side (1721.6 rad/s^2^) and front (1698.8 rad/s^2^).

### Quality assessment

Fifteen studies^16–27^^,^^29^^,^^32^^,^^33^ (HH, HD) were scored out of sixteen, and the other seven^14^^,^^15^^,^^28^^,^^30^^,^^31^^,^^34^^,^^35^ (MD, computer simulation) were scored out of thirteen. Of the former, five^16^^,^^17^^,^^25–27^ scored 16/16 (100%), six^18^^,^^20–22^^,^^24^^,^^29^ scored 15/16 (94%) and four^19^^,^^23^^,^^32^^,^^33^ scored 14/16 (88%). Of the latter, two^14^^,^^34^ scored 13/13 (100%), two^15^^,^^35^ scored 12/13 (92%), one^28^ scored 11/13 (85%) and two^30^^,^^31^ scored 10/13 (77%). All studies were considered to be of a moderate-to-high quality. A full scoring breakdown is attached in the supplementary material.

## Discussion

This review summarizes RA data from combat sport head impacts. RA was greater following direct strikes to the head compared to being thrown or taken down. Several impacts exceeded the proposed concussion threshold (4500 rad/s^2^) in boxing, taekwondo and MMA, with some also exceeding the proposed DAI and ASDH threshold (10 000 rad/s^2^). RA from impacts in judo and wrestling was almost always below injury thresholds. This suggests that athletes in boxing, taekwondo and MMA are at greater risk of TBI.

### Headgear

Headgear featured more often in striking sports (boxing, taekwondo). The physical designs used had been sanctioned by the appropriate governing bodies. Our findings show that head impacts in these sports can produce dangerously high RA values. Nevertheless, the use of headgear remains contentious.

Two studies (both MD) compared RA from different boxing impacts with and without headgear: every impact (hook punch, front, side, jaw) without headgear was in excess of 4500 rad/s.^2^ When used, RA was lowered to subthreshold levels for hook punches (1740 rad/s^2^), front (4335 rad/s^2^) and side impacts (4323 rad/s^2^). RA, however, albeit reduced, remained above the concussion threshold for impacts to the jaw (7173 rad/s^2^). Interestingly, a different HD study, which did not use headgear, measured RA from jaw impacts to be less (6896 rad/s^2^) than that of the aforementioned value.^19^ This might be explained by the jaw being poorly protected on certain designs, yet it would suggest that headgear does not always ensure safe RA values in boxing. In two other studies (both HD) using headgear, front impacts (6344–14 065 rad/s^2^) exceeded 4500 and 10 000 rad/s.^2^ Once more, if we compare this to another study (also HD), we see that RA measured with headgear (6344 rad/s^2^) was greater than without (5452 rad/s^2^).

Although the sample is small, the use of headgear appears to offer some benefit RA in boxing. However, it should be acknowledged that there is no clear and obvious pattern: the lowest RA in boxing was measured in a study that did not use headgear. Despite this published evidence, in 2013, the Amateur International Boxing Association (AIBA) banned headgear in all forms of competition, after a study identified that boxing without headgear lowered the chance of a fight ending in referee stoppage by 43%.^36^ Headgear is believed to increase the target area for opponents and decrease peripheral vision of the wearing athlete, and misconceptions surrounding the protective potential of headgear can result in more aggressive fighting styles that carry a greater risk of being hit, but these claims have been disputed.^37^^,^^38^ Impact measures such as RA do not appear to have been considered, and headgear (or equivalent) has also been shown to lower RA in sports such as American football and rugby union.^39^^,^^40^ This suggests that further research is necessary in boxing to assess the effect of such protective equipment on RA. Moreover, a recent systematic review concluded that the current evidence did not support the decision to prohibit the use of headgear in the sport.^41^

RA remained high in taekwondo despite athletes requiring headgear, and multiple impacts exceeded 10 000 rad/s^2^. Only one study recorded a subthreshold average RA (4455 rad/s^2^) on impact, and this would suggest that taekwondo athletes are most at risk of possibly severe TBI.^29^ For this reason, improvements to current headgear designs may be necessary to increase athlete safety. If changes to protective equipment were not to be sufficient, a revision of rules may be warranted. In judo, for example, throws carrying a high injury risk (kani-basami, kawazu-gake) are now forbidden,^42^

Headgear is not commonly worn in grappling sports (judo, wrestling), nor in MMA. One study documented that MMA athletes used headgear exclusively during sparring, with recorded RA (1766 rad/s^2^) much lower than when measured in competition (3773 rad/s^2^). In judo, a stronger emphasis is placed on correct technique to reduce the risk of TBI, and similar RA values were obtained from throws irrespective of whether or not headgear was worn. Amateur wrestlers are required to wear headgear in some countries, but not in international senior competition. Even so, this is primarily for ear protection, and such headgear offers no recognized protection against TBI.

### Associated injury/LOC

RA resulting in concussion was recorded in two MMA studies (both HH). In concussive impacts (7560.8–7561 rad/s^2^), RA was greater than in those causing no injury (5055.7–7069.5 rad/s^2^). A similar trend was observed in boxing, where RA from LOC punches (11279.5 rad/s^2^) was significantly higher than non-LOC punches (6145.5 rad/s^2^).

Non-injury–causing impacts, in striking sports particularly, easily exceed proposed thresholds, and this includes impacts that occur during lower-intensity and non-competitive (i.e. sparring) environments. One study found RA during MMA sparring to be above the concussion threshold (5055.7 rad/s^2^). While sparring is not directly applicable to real fighting, RA can remain high and impacts are potentially injurious. There is a significant risk of TBI to athletes in competitive and non-competitive settings, who, critically, may not be visibly incapacitated. It is vital that those displaying signs or symptoms consistent with TBI are immediately removed from play and evaluated by a medical professional.

### Ukemi (breakfall) and under-mats

Judo players are taught to perform ukemi (breakfall) when landing to minimize the risk of TBI. Correct ukemi technique prevents direct head contact with the mat and can therefore lower RA after being thrown. We see its value when comparing athlete and dummy studies, given that only athletes can execute ukemi. RA from osoto-gari in HH studies (679.4–693.2 rad/s^2^) was lower than that in HD studies (3315–5081 rad/s^2^), and this is also the case for ouchi-gari and seoi-nage throws. Judo players should continue to learn optimal technique before practising throws with a high risk of injury. Under-mats may help to prevent injury in judo, but the increased RA with their use in ouchi-gari raises questions about their overall efficacy.

### Clinical implications and future work

The findings of the present systematic review should inform future research and highlight areas in combat sports where action can be taken. Although all studies were of a good quality, a small number of studies were less than optimal, and the heterogeneity of the available data made it challenging to formulate sport-specific recommendations. We acknowledge that differences between individual methodologies (HH, HD, MD), along with the specific testing conditions of each study, will have influenced the data produced by the various investigations. In a laboratory (HD, MD) setting, this includes athletes impacting dummies with various amounts of force, and mechanical devices impacting these devices at fixed velocities. Equally, there are physical variables in entirely human (HH) studies that cannot be replicated in the laboratory. Fatigue, in particular, negatively affects reaction times and consistency (increasing susceptibility to TBI) but reduces the magnitude of impacts delivered.^43^^,^^44^ Altogether, this makes it difficult to apply laboratory data to physical combat situations, and comparing data across study types must be done with caution.

We acknowledge that some of the investigations included in the present study are laboratory based. Laboratory RA data demonstrate the capability of combat sport athletes to inflict catastrophic and life-threatening damage in a practice, not competition, situation from one single impact. For this reason, it would be unethical to replicate such studies with human participants. Laboratory data do not mirror competitive fighting, but these results highlight impacts in these arguably lower-intensity situations can cause TBI: RA likely increases in higher-intensity environments.

The possible error associated with the delivery of mechanical impacts should also be discussed. The Hybrid III ATD headform, for instance, overestimates RA by 8%.^45^ Moreover, these impacts were delivered linearly and do not represent the rotational body motion of athletes. Above all, the thresholds discussed in this review are only some of those that exist in literature: evidently, different thresholds would change the way in which our results are interpreted. RA thresholds should not be used in the absence of physical assessment to diagnose TBI. For example, osoto-gari is strongly associated with ASDH in judo, and a maximum RA of 5081.3 rad/s^2^ is evidently below the proposed injury threshold. This may seem unsurprising, as we know composite variables to be more sensitive in predicting TBI. However, lower RA causes less structural brain damage; hence, RA can continue to offer direction for safety changes in combat sports.^46^

Meanwhile, it is essential that governing bodies strive to minimize RA produced from head impacts, and we recommend that future prospective research involving combat sport athletes is performed to achieve this. We encourage the use of appropriate instrumented equipment to allow for the collection of *in vivo* RA data.^45^^,^^47^ Head Impact Telemetry (HIT) is an example of a validated system used in sport and can be incorporated into headgear and mouthguards to collect information about RA.^45^ Other systems have enabled the development of instrumented headbands, skullcaps and skin patches, but currently lack validation.

Ultimately, the diagnosis of TBI in combat sports is facilitated by the presence of on-site physicians. Athletes are monitored carefully in the professional setting, but most compete in environments where trained medical personnel are not routinely available, and assessment is left in the hands of athletes and coaches. Knowledge of TBI in these groups remains poor, and it is estimated that 40% of athletes immediately return to play after a suspected TBI.^48^ There is a need for education programmes to be made available in combat sports and it is unfair to assume a degree of clinical competency from coaches or officials. RA can help to contextualize TBI in combat sports as the relevant techniques can be understood by athletes, coaches and researchers alike. Thus, RA can be used to assess the risk of training activities and ensure proper planning of training to offer adequate rest between sessions. Sessions should also follow gradual loading and start with lower RA activities before moving on to higher RA activities to reduce risk of TBI. This would arguably be of most benefit in MMA, given the differences in RA between strikes and throws. A recent study also found that periodization of training load was largely absent amongst MMA athletes.^49^ RA, along with other measures of training intensity, may help to prioritize player safety, prevent overtraining and ensure that the enjoyment of combat sport is maintained.^50^

Finally, the cumulative effect of subthreshold impacts in combat sports should not be forgotten. Growing evidence links the effects of repeated head impacts to dementia, depression and chronic traumatic encephalopathy (CTE).^51^ There is also a greater possibility of musculoskeletal injury following TBI in sport, and neuromuscular risk factors should be considered when determining return to play.^52^ The mismanagement of these impacts increases the short-term risk of severe TBI.^53^

### Limitations

There are certainly some limitations to the present systematic review. Firstly, we collated RA from studies presenting peak, and average peak, values. In those measuring average peak (±SD) RA, the entire range was not consistently available; thus, we are not able to comment on this any further. Secondly, we did not consider the effect of any other biomechanical variables (i.e. impact velocity, impulse time), albeit beyond the scope of the present work. Lastly, the characteristics of athletes in studies involving human participants (age, sex, height, weight, experience level) were not presented in the original studies. This is also likely to influence our data.

## Conclusion

Head impacts in combat sports produce significant RA, and various thresholds for different types of TBI have been proposed. Direct head impacts in striking sports produced greater RA values than impacts from throws or takedowns in grappling sports and appear to put athletes at greater risk of TBI. Differences in study methodologies and testing conditions meant that accurate sport-specific conclusions could not be drawn. However, the present systematic review serves as a reminder that dangerously high RA values can be generated even in non-competitive environments (laboratory, sparring). The use of headgear in boxing lowered RA, allowing RA to reach subthreshold levels. Similarly, modifications to headgear design in taekwondo may be necessary given the substantial RA generated from rotating kicks. RA remains a useful predictor of TBI, as lower values are associated with less structural brain damage. Non-injury–causing impacts can exceed TBI thresholds and therefore carry a real risk of injury, and it is imperative that athletes showing signs or symptoms consistent with TBI are removed from play and medically assessed. Greater awareness of RA in combat sports affords coaches the opportunity to risk stratify training activities and ensure appropriate rest periods between sessions. Suitable educational tools must also be made available to athletes and coaches of all abilities. Future prospective research should measure RA from real-life impacts and document associated injury outcomes in order to further our understanding of TBI in combat sports. For the time being, sporting governance must strive to minimize RA from head impacts to maximize athlete safety.

## Conflicts of interest statement

The authors have no potential conflicts of interest.

## Funding

No external sources of funding.

## Contributors

KSL, NM and VK contributed to study design. KSL drafted the manuscript and NM, VK and NM contributed to writing of the manuscript. All authors revised the draft manuscript and approved the final version.

## Patient consent

Not required.

## Ethical approval

Not required.

## Data availability statement

No new data were generated or analyzed in support of this review.

## Supplementary Material

Supplementary_Material_ldac002

## References

[1] McKee AC , Daneshvar DH. The neuropathology of traumatic brain injury. Handb Clin Neurol., 2015;127:45–66. doi: 10.1016/B978-0-444-52892-6.00004-0 PMC4694720 25702209

[2] Galgano M , Toshkezi G, Qiu X, et al. Traumatic brain injury: current treatment strategies and future endeavors. Cell Transplant 2017;26:1118–30. 10.1177/0963689717714102. 28933211 PMC5657730

[3] Thurman DJ , Branche CM, Sniezek JE. The epidemiology of sports-related traumatic brain injuries in the United States: recent developments. J Head Trauma Rehabil 1998;13:1–8. 10.1097/00001199-199804000-00003. 9575252

[4] Jayarao M , Chin LS, Cantu RC. Boxing-related head injuries. Phys Sportsmed 2010;38:18–26. 10.3810/psm.2010.10.1804. 20959692

[5] Kleiven S . Why most traumatic brain injuries are not caused by linear acceleration but skull fractures are. Front Bioeng Biotechnol 2013;1. 10.3389/fbioe.2013.00015. PMC4090913 25022321

[6] Zhang J , Yoganandan N, Pintar FA, et al. Role of translational and rotational accelerations on brain strain in lateral head impact. Biomed Sci Instrum. 2006;464:501–6. 16817658

[7] Piedade SR , Hutchinson MR, Ferreira DM, et al. The management of concussion in sport is not standardized. A systematic review. J Saf Res 2021;76:262–8. 10.1016/j.jsr.2020.12.013. 33653558

[8] Harmon KG , Drezner JA, Gammons M, et al. American Medical Society for Sports Medicine position statement: concussion in sport. Br J Sports Med 2013;47:15–26. 10.1136/bjsports-2012-091941. 23243113

[9] Depreitere B , Van Lierde C, Vander Sloten J, et al. Mechanics of acute subdural hematomas resulting from bridging vein rupture. J Neurosurg 2006;104:950–6. 10.3171/jns.2006.104.6.950. 16776340

[10] Ommaya AK , Goldsmith W, Thibault L. Biomechanics and neuropathology of adult and paediatric head injury. Br J Neurosurg 2002;16:220–42. 10.1080/02688690220148824. 12201393

[11] Kerr ZY , Campbell KR, Fraser MA, et al. Head impact locations in U.S. high school boys’ and girls’ soccer concussions, 2012/13-2015/16. J Neurotrauma 2019;36:2073–82. 10.1089/neu.2017.5319. 29092652

[12] Downes MJ , Brennan ML, Williams HC, et al. Development of a critical appraisal tool to assess the quality of cross-sectional studies (AXIS). BMJ Open 2016;6:e011458. 10.1136/bmjopen-2016-011458. PMC5168618 27932337

[13] Bhalerao S , Kadam P. Sample size calculation. Int J Ayurveda Res 2010;1:55. 10.4103/0974-7788.59946. 20532100 PMC2876926

[14] Bartsch AJ , Benzel EC, Miele VJ, et al. Boxing and mixed martial arts: preliminary traumatic neuromechanical injury risk analyses from laboratory impact dosage data. J Neurosurg 2012;116:1070–80. 10.3171/2011.12.JNS111478. 22313361

[15] Beckwith JG , Chu JJ, Greenwald RM. Validation of a noninvasive system for measuring head acceleration for use during boxing competition. J Appl Biomech 2007;23:238–44. 10.1123/jab.23.3.238. 18089922

[16] Fife GP , O’sullivan DM, Lee SY. Rotational and linear head accelerations from taekwondo kicks and punches. J Sports Sci 2018;36:1461–4. 10.1080/02640414.2017.1398406. 29099672

[17] Hecimovich M , King D, Garrett T. Accelerometric analysis of head impacts in amateur wrestling: an exploratory analysis. Int J Wrestl Sci 2016;6:117–26. 10.1080/21615667.2017.1315842.

[18] Stojsih S , Boitano M, Wilhelm M, et al. A prospective study of punch biomechanics and cognitive function for amateur boxers. Br J Sports Med 2010;44:725–30. 10.1136/bjsm.2008.052845. 19019907

[19] Viano DC , Casson IR, Pellman EJ, et al. Concussion in professional football: comparison with boxing head impacts - part 10. Neurosurgery 2005;57:1154–70. 10.1227/01.NEU.0000187541.87937.D9. 16331164

[20] Walilko TJ , Viano DC, Bir CA. Biomechanics of the head for Olympic boxer punches to the face. Br J Sports Med 2005;39:710–9. 10.1136/bjsm.2004.014126. 16183766 PMC1725037

[21] Doan BK , Heaton KJ, Self BP, et al. Quantifying head impacts and neurocognitive performance in collegiate boxers. J Sports Sci Published online December 2021;20:1–9. 10.1080/02640414.2021.2001175. 34930100

[22] Jansen AE , McGrath M, Samorezov S, et al. Characterizing head impact exposure in men and women during boxing and mixed martial arts. Orthop J Sports Med 2021;9:232596712110598. 10.1177/23259671211059815. PMC8664317 34901294

[23] Hitosugi M , Murayama H, Motozawa Y, et al. Biomechanical analysis of acute subdural hematoma resulting from judo. Biomed Res (Japan) 2014;35:339–44. 10.2220/biomedres.35.339. 25355441

[24] Ishikawa Y , Anata K, Hayashi H, et al. Effects of different throwing techniques in judo on rotational acceleration of Uke’s head. Int J Sport Health Sci 2018;16:173–9. 10.5432/ijshs.201713.

[25] Murayama H , Hitosugi M, Motozawa Y, et al. Ukemi technique prevents the elevation of head acceleration of a person thrown by the judo technique osoto-gari. Neurol Med Chir 2020;60:307–12. 10.2176/nmc.oa.2020-0043. PMC7301129 32404577

[26] Murayama H , Hitosugi M, Motozawa Y, et al. Biomechanical analysis of the head movements of a person thrown by the judo technique seoi-nage. Neurol Med Chir 2020;60:101–6. 10.2176/nmc.oa.2019-0206. PMC7040429 31866665

[27] Murayama H , Hitosugi M, Motozawa Y, et al. Rotational acceleration during head impact resulting from different judo throwing techniques. Neurol Med Chir 2014;54:374–8. 10.2176/nmc.oa.2013-0227. PMC4533442 24477065

[28] O’Sullivan DM , Fife GP, O’Sullivan DM, et al. Impact attenuation of protective boxing and taekwondo headgear. Eur J Sport Sci 2016;16:1219–25. 10.1080/17461391.2016.1161073. 26999564

[29] O’Sullivan DM , Fife GP. Biomechanical head impact characteristics during sparring practice sessions in high school taekwondo athletes. J Neurosurg Pediatr 2017;19:662–7. 10.3171/2017.1.PEDS16432. 28387642

[30] Boroushak N , Eslami M, Kazemi M, et al. The dynamic response of the taekwondo roundhouse kick to head using computer simulation. Ido Movement for Culture 2018;18:54–60. 10.14589/ido.18.2.8.

[31] Boroushak N , Khoshnoodi H, Rostami M. Investigation of the head’s dynamic response to boxing punch using computer simulation. Montenegrin J Sports Sci Med 2021;10:31–5. 10.26773/mjssm.210305.

[32] Tiernan S , Meagher A, O’Sullivan D, et al. Concussion and the severity of head impacts in mixed martial arts. Proc Inst Mech Eng H J Eng Med 2020;234:1472–83. 10.1177/0954411920947850. PMC10330964 32799750

[33] Tiernan S , Meagher A, O’Sullivan D, et al. Finite element simulation of head impacts in mixed martial arts. Comput Methods Biomech Biomed Engin. Published online October 2020;24:1–11. 10.1080/10255842.2020.1826457. PMC10330963 33017178

[34] Cournoyer J , Hoshizaki TB. Head dynamic response and brain tissue deformation for boxing punches with and without loss of consciousness. Clin Biomech (Bristol, Avon) 2019;67:96–101. 10.1016/j.clinbiomech.2019.05.003. 31082637

[35] McIntosh AS , Patton DA. Boxing headguard performance in punch machine tests. Br J Sports Med 2015;49:1108–12. 10.1136/bjsports-2015-095094. 26175022

[36] Loosemore MP , Butler CF, Khadri A, et al. Use of head guards in AIBA boxing tournaments—a cross-sectional observational study. Clin J Sport Med 2017;27:86–8. 10.1097/JSM.0000000000000322. 27116592

[37] Sethi NK . In response to: use of head guards in AIBA boxing tournaments-a cross-sectional observational study. Clin J Sport Med: official journal of the Canadian Academy of Sport Medicine 2018;28:e1. 10.1097/JSM.0000000000000401. 27740948

[38] Menger R , Menger A, Nanda A. Rugby headgear and concussion prevention: misconceptions could increase aggressive play. Neurosurg Focus 2016;40:E12. 10.3171/2016.1.FOCUS15615. 27032915

[39] Zuckerman SL , Reynolds BB, Yengo-Kahn AM, et al. A football helmet prototype that reduces linear and rotational acceleration with the addition of an outer shell. J Neurosurg 2019;130:1634–41. 10.3171/2018.1.JNS172733. PMC6289811 29957115

[40] Ganly M , McMahon JM. New generation of headgear for rugby: impact reduction of linear and rotational forces by a viscoelastic material-based rugby head guard. BMJ Open Sport Exerc Med 2018;4:464. 10.1136/bmjsem-2018-000464. PMC6307573 30622730

[41] Tjønndal A , Haudenhuyse R, de Geus B, et al. Concussions, cuts and cracked bones: a systematic literature review on protective headgear and head injury prevention in Olympic boxing. Eur J Sport Sci. Published online February 19 2021;1–13. 10.1080/17461391.2021.1872711. 33607924

[42] Vacca L , Rosso V, Gastaldi L. Risk assessment in different judo techniques for children and adolescent athletes. Proc Inst Mech Eng H J Eng Med 2020;234:686–96. 10.1177/0954411920915589. 32292102

[43] Sant’Ana J , Franchini E, da Silva V, et al. Effect of fatigue on reaction time, response time, performance time, and kick impact in taekwondo roundhouse kick. Sports Biomech. 2017;16:201–9 doi: 10.1080/14763141.2016.1217347. 27592682

[44] Pavelka R , Třebický V, Fialová JT, et al. Acute fatigue affects reaction times and reaction consistency in mixed martial arts fighters. PLoS One 2020;15:e0227675. 10.1371/JOURNAL.PONE.0227675. 32004350 PMC6994193

[45] Patton DA . A review of instrumented equipment to investigate head impacts in sport. Appl Bionics Biomech 2016;1–16. 10.1155/2016/7049743. PMC4993933 27594780

[46] Mainwaring L , Ferdinand Pennock KM, Mylabathula S, et al. Subconcussive head impacts in sport: a systematic review of the evidence. Int J Psychophysiol 2018;132:39–54. 10.1016/j.ijpsycho.2018.01.007. 29402530

[47] Camarillo DB , Shull PB, Mattson J, et al. An instrumented mouthguard for measuring linear and angular head impact kinematics in american football. Ann Biomed Eng 2013;41:1939–49. 10.1007/s10439-013-0801-y. 23604848 PMC3954756

[48] Bennett LL , Arias JJ, Ford PJ, et al. Concussion reporting and perceived knowledge of professional fighters. Phys Sportsmed 2019;47:295–300. 10.1080/00913847.2018.1552481. 30479188 PMC6536361

[49] Kirk CI , Langan-Evans C, Clark DR, et al. Quantification of training load distribution in mixed martial arts athletes: a lack of periodisation and load management. PLoS One. 2021;16:e0251266. Published 2021 May 10. 10.1371/journal.pone.0251266. PMC8109772 33970947

[50] Ouergui I , Franchini E, Selmi O, et al. Relationship between perceived training load, well-being indices, recovery state and physical enjoyment during judo-specific training. Int J Environ Res Public Health 2020;17:7400. 10.3390/IJERPH17207400. PMC7650691 33050671

[51] McKee AC , Alosco ML, Huber BR. Repetitive head impacts and chronic traumatic encephalopathy. Neurosurg Clin N Am 2016;27:529–35. 10.1016/j.nec.2016.05.009. 27637402 PMC5028120

[52] Kakavas G , Malliaropoulos N, Blach W, et al. Ball heading and subclinical concussion in soccer as a risk factor for anterior cruciate ligament injury. J Orthop Surg Res 2021;16(1):1–4. doi:10.1186/S13018-021-02711-Z. 34538251 10.1186/s13018-021-02711-z PMC8451147

[53] Yokota H , Ida Y. Acute subdural hematoma in a judo player with repeated head injuries. World Neurosurg 2016;91:671.e1–3. 10.1016/j.wneu.2016.03.101. 27060521

